# A Phase 2 evaluation of a new flavored peg and sulfate solution compared to an over-the-counter laxative, peg and sports drink bowel preparation combination

**DOI:** 10.1186/s12876-023-03069-8

**Published:** 2023-12-11

**Authors:** Gregory Wiener, Peter Winkle, John D. McGowan, Mark vB. Cleveland, Jack A. Di Palma

**Affiliations:** 1 GW Research, Inc, Chula Vista, CA USA; 2 CenExel ACT Anaheim, Chula Vista, Anaheim, CA USA; 3 Braintree Laboratories, Inc, Braintree, MA USA; 4 Frederick P. Whiddon College of Medicine, University of South Alabama, 75 University Blvd., South Mobile, AL 36688 USA

**Keywords:** Bowel prepration, Colonoscopy, Laxative, Sports drink, Polyethylene glycol

## Abstract

**Background:**

Acceptability and tolerance of bowel preparation is critical to overcome patient hesitancy in undergoing colon cancer screening and surveillance colonoscopy. To improve patient experience, a new sports drink-flavored bowel preparation containing polyethylene glycol (PEG) and sulfate salts (FPSS) was developed to provide a similar experience to a commonly used but not United States Food and Drug Administration (FDA) approved PEG and sports drink bowel preparation (PEG-SD), while also achieving improved cleansing efficacy.

**Methods:**

This FPSS preparation, approved by the FDA in June 2023, was evaluated in a non-randomized Phase 2 study in which 40 patients requiring colonoscopy were prepared with FPSS and 20 with PEG-SD.

**Results:**

Overall cleansing success was high with FPSS based on unblinded local endoscopist assessment (93%) and blinded central reading (97%), exceeding PEG-SD which achieved success rates of 84% (local read), 74% and 68% (blinded central reads). Similar differences favoring FPSS were seen for excellent preparations and cleansing success by colon segment as rated by local endoscopists. Both preparations were well-tolerated, with 93% of FPSS patients rating their preparation as Tolerable to Very Easy to consume, compared to 100% of PEG-SD. Patients who had previously taken a preparation for colonoscopy found FPSS and PEG-SD better than their prior preparation (73% and 70%, respectively) and nearly all would request their assigned study preparation again in the future. About two thirds of FPSS patients agreed that the preparation tasted similar to a sports drink.

**Conclusion:**

The new sports drink-like flavored preparation compares favorably to PEG-SD for bowel cleansing efficacy while achieving similar patient satisfaction. The study was registered at www.clinicaltrials.gov (NCT03328507) on 01/11/2017.

## Introduction

Despite improvements in prescription bowel preparation technology, a significant proportion of colonoscopy preparations offered to patients are not approved by the FDA, but rather are over-the-counter laxative combinations [1]. This is likely because bowel preparation is repeatedly cited as the worst part of the colonoscopy experience and an impediment to recommended surveillance [2]. To combat prep hesitancy on the part of patients, many physicians have adopted an unapproved bowel preparation for colonoscopy that consists of an over-the-counter polyethylene glycol (PEG-3350) laxative combined with a “sports drink” (PEG-SD) such as Gatorade® [3]. A stimulant laxative (e.g., bisacodyl) is frequently included in this regimen. This combination contains electrolytes and sugar in addition to PEG, however the electrolyte content, particularly sodium, is much lower than in most formulated bowel preparations. As a result, PEG-SD is an osmotically and electrolyte unbalanced formulation. Studies of its effects on fluid and electrolyte balance have shown that in normal volunteers PEG-SD results in body absorption of large volumes of water and substantial losses of sodium. In a study by Matro et al. [3], patients were randomized to receive PEG-SD (n = 180) or polyethylene glycol with electrolytes and ascorbic acid (PEG-EA), an FDA approved bowel preparation (MOVIPREP^®,^ Salix Pharmaceuticals, Bridgewater, NJ) (n = 184) and collected clinical chemistry data at baseline and on the day of colonoscopy. The incidence of hyponatremia (serum sodium < 136 mmol/L) was higher in the PEG-SD group (3.9%) versus the PEG-EA treated group (2.2%). Following preparation, statistically significant changes from baseline were associated with PEG-SD for serum sodium, potassium and chloride.

Studies by Walker et al. [4] have demonstrated that PEG-SD produces less cleansing diarrhea (2.0 L) than other FDA approved bowel preparations (e.g., oral sulfate solution = 3.3 L, PEG-EA = 3.0 L) which helps to explain reports by Matro et al. [3], that PEG-SD bowel preparation produces inferior cleansing compared to other FDA approved preparations. Nevertheless, physicians continue to recommend PEG-SD because patient perception and completion rates are better relative to high-volume 4-liter options [1, 3, 5, 6].

To provide healthcare practitioners and patients the positive attributes of PEG-SD (e.g., taste, low volume) in an electrolyte-balanced formulation, a new sports drink-like flavored PEG and sulfate solution (FPSS, SUFLAVE^®^, Braintree Laboratories, Inc.) was developed and recently approved by the FDA. This preparation does not require the addition of bisacodyl (a harsh stimulant laxative associated with reports of ischemic colitis) to improve efficacy. The FDA requires all approved prescription bowel preparations to warn against concurrent use of stimulant laxatives due to the risk of mucosal ulceration and ischemic colitis. In Phase 1 development studies, FPSS produced 3.5 L of cleansing diarrhea, comparable to oral sulfate solution [4]. This report describes a preliminary evaluation of the safety, efficacy and tolerance of FPSS compared to PEG-SD for bowel preparation prior to colonoscopy in adult patients.

## Methods

This was an open-label, active-controlled, sequential-cohort study in adult patients undergoing colonoscopy for colorectal cancer screening and surveillance or for diagnostic purposes. This study was conducted at 4 U.S. endoscopy centers and approved by Aspire, Institutional Review Board and registered on www.clinicaltrials.gov (NCT03328507) on 01/11/2017. The investigation conformed with the principles outlined in the Declaration of Helsinki. Subjects with known or suspected ileus, severe ulcerative colitis, gastrointestinal obstruction, gastric retention, bowel perforation, toxic colitis or megacolon or who had previous significant gastrointestinal surgeries were excluded. Also excluded were patients with uncontrolled pre-existing electrolyte abnormalities, uncontrolled hypertension, known severe hepatic insufficiency (Child Pugh C), and subjects with cardiac insufficiency (NYHA Functional Classifications 3 or 4).

A screening visit was performed for eligible patients within 30 days prior to their scheduled colonoscopy which included routine blood chemistry. In this adaptive design study conducted over a two-year period, twenty subjects were planned to take the PEG-SD control, with various experimental formulations concurrently and subsequently evaluated (not discussed here) with 40 subjects ultimately planned to take the FPSS to-be-marketed formulation. Preparation administration was not randomized.

On the day prior to colonoscopy, subjects were allowed a low residue breakfast followed by clear liquids until the colonoscopy was completed the following day. Both preparations were administered using the American College of Gastroenterology recommended split-dose (PM/AM) regimen. Subjects assigned to FPSS reconstituted the one-liter dose with water on the evening prior to colonoscopy and consumed 8 oz of solution every 15 min until complete. A second one-liter dose was taken the morning of colonoscopy. FPSS subjects drank an additional 16oz of water following each preparation dose. Subjects assigned to PEG-SD received two 5 mg over-the-counter bisacodyl laxative tablets, two 32-ounce bottles of Lemon-Lime flavored sports drink (Gatorade; PepsiCo, Inc., Chicago, IL, USA), and two 119 g bottles of Polyethylene glycol 3350 (PEG 3350). Subjects were instructed to take the bisacodyl at approximately 3PM on the day prior to colonoscopy. Subjects then mixed one bottle of PEG 3350 with one bottle of Gatorade and consumed 8 oz every 15 min until complete, along with additional water. A second one-liter dose was taken the morning of colonoscopy.

Subjects returned to the clinic the day of colonoscopy. Patients were queried for occurrence of adverse events, given a physical examination, and a blood sample was taken for routine chemistry. Colonoscopies were performed by unblinded endoscopists according to the site’s standard procedures. Colon cleanliness was assessed following completion of the exam using a 4-point scale (Table 1) previously used in Phase 3 studies of approval bowel preparations [7]. Cleansing scores of “good” and “excellent” were considered successful and scores of “fair” and “poor” were considered failures (the primary endpoint). In addition, quality indicators of cecal intubation and assessment of clinically adequate cleansing were collected. In addition to local endoscopist grading, blinded central reading was performed for each colonoscopy video. All videos were rated by the same two blinded endoscopists.

**Table 1:** Bowel Preparation Cleansing Scale

Score	Description
Poor	Large amounts of fecal residue, additional bowel preparation required
Fair	Enough feces even after washing and suctioning to prevent clear visualization of the entire colonic mucosa.
Good	Feces and fluid requiring washing and suctioning, but still achieves clear visualization of the entire colonic mucosa.
Excellent	No more than small bits of feces/fluid which can be suctioned easily; achieves clear visualization of the entire colonic mucosa.

Prior to their procedure, all patients were asked to complete a questionnaire which included questions related to preparation satisfaction, including: How easy or difficult was it to consume the study preparation (Very Difficult to Very Easy)?; Please describe your overall experience with the bowel preparation (Bad to Excellent); How did this bowel preparation experience compare to your prior experiences (Worse to Better)?; Would you ask your doctor for this preparation again if you need another colonoscopy in the future? (Yes or No); Would you refuse the same preparation again if it were to be prescribed to you in the future? (Yes or No). In addition, FPSS patients were asked the following: Please rate your feelings about the aftertaste of the preparation (Very Pleasant to Very Unpleasant), and; To what degree do you believe the product tastes similar to a sports drink? (Agree Extremely to Disagree Extremely).

## Results

**Fig. 1:**
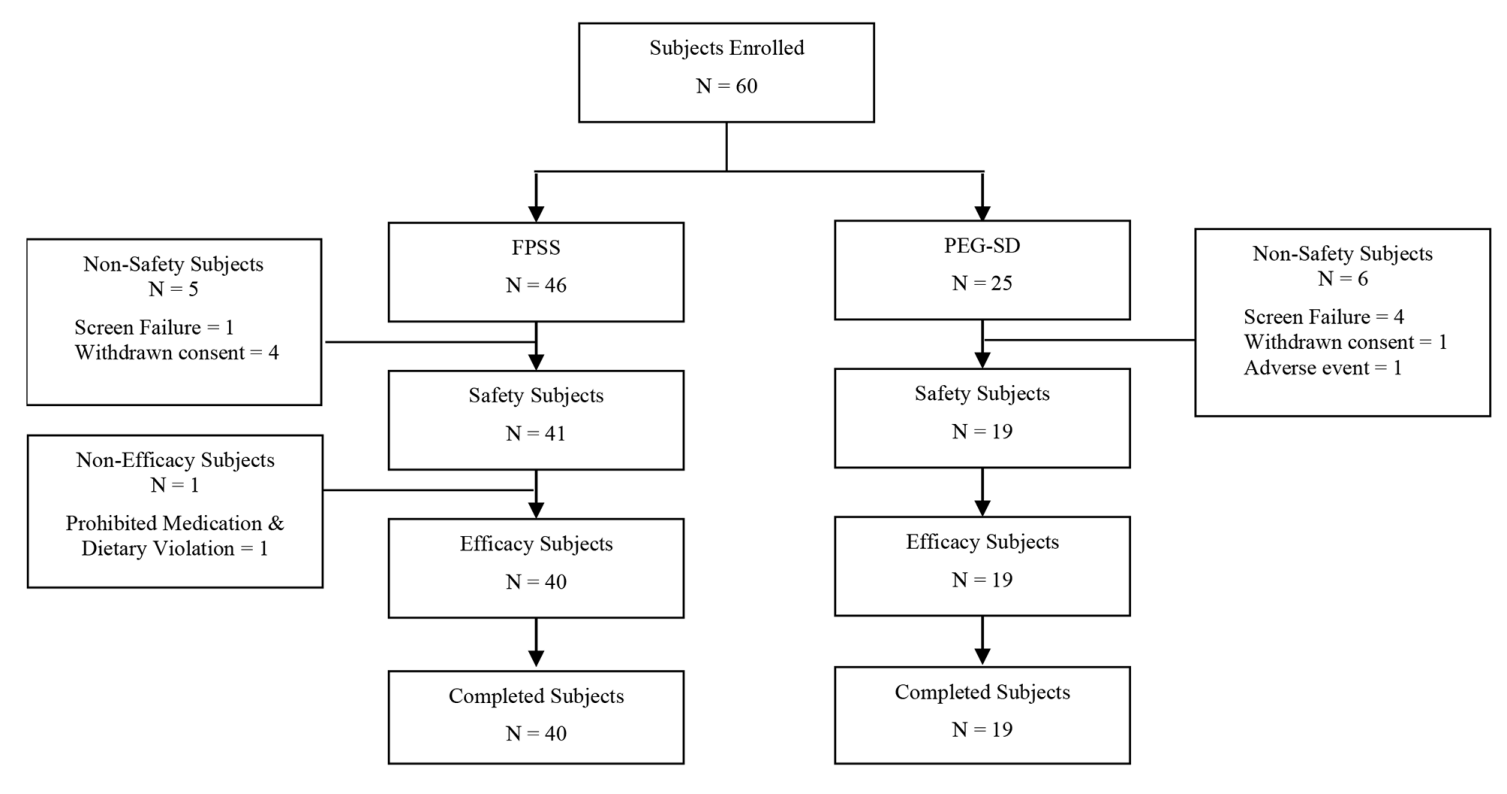
Patient Disposition

Forty-one patients received FPSS and 19 patients received PEG-SD (Fig. 1).These patients were enrolled from 3 centers in Southern California and were demographically similar with respect to gender, ethnicity and race (Table 2). The average age of PEG-SD patients was about 10 years younger than FPSS patients (PEG-SD 49.7 years; FPSS 59.0 years).

**Table 2:** Patient Demographics

	FPSS n (%)	PEG-SD n (%)
Mean Age (SD)	59.0 (10.3)	49.7 (15.7)
Age > 65 yrs	12 (29.3)	2 (10.5)
Male	16 (39.0)	7 (36.8)
Female	25 (61.0)	12 (63.2)
African American	4 (9.8)	1 (5.3)
Caucasian	35 (85.3)	18 (94.8)
Other race	2 (4.9)	0

^1^Demographics based on Safety Population

^2^Percentages based on subjects who discontinued in each treatment group

Table 3 shows that overall preparation success was higher in association with FPSS than with PEG-SD. This was evident in ratings by both local endoscopists (93% vs. 84%) and blinded central reviewers whose ratings for preparation success tended to be higher for FPSS (97%) and much lower for PEG-SD (average of 71%). The cleansing success of FPSS is consistent with reports of highly effective bowel preparations (e.g., SUPREP) using the same grading scale. FPSS also produced a higher rate of excellent preparations compared to PEG-SD (local endoscopist ratings: 74% versus 53%) based on local endoscopist ratings. Ratings of individual colon segments by the local endoscopists were consistent with overall cleansing success, with the largest difference seen in the right colon (95% FPSS cleansing success vs. 68% for PEG-SD, Table 4).

**Table 3:** Overall Cleansing Assessment

	FPSS (N = 40)	PEG-SD (N = 19)
Local Endoscopist		
Overall Cleansing Success		
Success	37 (92.5)	16 (84.2)
Failure	3 (7.5)	3 (15.8)
Grade (n %)		
Excellent	29 (74.4)	10 (52.6)
Good	8 (20.5)	6 (31.6)
Fair	2 (5.1)	2 (10.5)
Poor	0	1 (5.3)
Blinded Central Review		
Overall Cleansing Success		
Central Reader 1	38 (97.4)^1^	14 (73.7)
Central Reader 2	38 (97.4)^1^	13 (68.4)

^1^p-value < 0.05 for comparison of FPSS to PEG-SD

**Table 4:** Segmental Cleansing Score by Grade

	Grade (n %)	FPSS (N = 40)	PEG-SD (N = 19)
Proximal Colon Segment	Poor	0	1 (5.3)
	Fair	2 (5.1)	5 (26.3)
	Good	12 (30.8)	4 (21.1)
	Excellent	25 (64.1)	9 (47.4)
Mid Colon Segment	Poor	0	0
	Fair	1 (2.6)	2 (10.5)
	Good	7 (17.9)	7 (36.8)
	Excellent	31 (79.5)	10 (52.6)
Distal Colon Segment	Poor	0	1 (5.3)
	Fair	2 (5.1)	1 (5.3)
	Good	8 (20.5)	7 (36.8)
	Excellent	29 (74.4)	10 (52.6)

Secondary endpoints were largely equivalent between the preparations, with no important differences observed for procedure duration (FPSS: 11.6 min, PEG-SD: 10.7 min), volume of irrigation water used (FPSS: 77.4 ml, PEG-SD: 61.1 ml), and clinically adequate cleansing (FPSS: 100%, PEG-SD: 94.7%). Cecal intubation was achieved in all colonoscopies.

Treatment-emergent adverse events were consistent with expected bowel preparation symptoms and were generally mild or moderate in severity, with the exception of one patient in each group who reported severe nausea and vomiting. Frequencies of solicited preparation symptoms of nausea, vomiting, abdominal cramping and bloating were all less than 20% for FPSS, with only 3 subjects experiencing vomiting (7%). One FPSS patient had a report of worsening of pre-existing bradyarrhythmia and one PEG-SD patient experienced mild orthostatic hypertension. Both events were considered to be “possibly” related to preparation.

Changes in serum chemistry parameters as a result of colonoscopy preparation were small and not clinically significant for either preparation group. One FPSS patient experienced hypocalcemia and one PEG-SD patient experienced hypernatremia.

Results from the patient satisfaction questionnaire are displayed below in Table 5. Both preparations were well tolerated with respect to ease of use, with 93% of FPSS patients rating their preparation as Tolerable to Very Easy to consume, compared to 100% for PEG-SD. More PEG-SD patients tended to rate their preparation as Easy or Very Easy to consume. Overall experience ratings favored FPSS, with 98% of FPSS patients rating their experience as Excellent or Good compared to 74% for PEG-SD. Each preparation was favored over patients’ prior bowel prep experience (70–73%). Ratings of acceptance for future colonoscopy were high, with ≥ 95% of patients indicating they would request the same preparation (FPSS – 98%, PEG-SD – 95%). Lastly, about two thirds (68%) of FPSS subjects agreed that the preparation tasted similar to a sports drink and 85% of FPSS patients rated the aftertaste as Neutral to Very Pleasant.

**Table 5:** Patient Satisfaction Questionnaire

Question	FPSS (N = 40)	PEG-SD (N = 19)
Ease of Consuming Prep (n %)		
Very Easy	11 (27.5)	9 (47.4)
Easy	11 (27.5)	6 (31.6)
Tolerable	15 (37.5)	4 (21.1)
Difficult	3 (7.5)	0
Very Difficult	0	0
Overall Experience (n %)		
Excellent	13 (32.5)	4 (21.1)
Good	26 (65.0)	10 (52.6)
Fair	1 (2.5)	4 (21.1)
Poor	0	1 (5.3)
Bad	0	0
Comparison to Prior Experience (n %)		
Better	11 (73.3)	7 (70.0)
Same	3 (20.0)	1 (10.0)
Worse	1 (6.7)	2 (20.0)
NA (no prior colonoscopy)	25	10
Would You Request Again? (n %)		
Yes	39 (97.5)	18 (94.7)
Would You Refuse?		
No	38 (95.0)	17 (89.5)

## Conclusions

This Phase 2 pilot study demonstrates that the cleansing efficacy of FPSS (> 90% success) appears to be similar to FDA-approved bowel preparations. Furthermore, it also suggests that the lower stool output reported for PEG-SD impacts visualization during colonoscopy, with PEG-SD achieving lower overall success ratings based on both local (84%) and blinded central reader review (average of 71%). However, local endoscopists were ultimately able to achieve an adequate exam for PEG-SD in 95% of cases and 100% for FPSS, with all procedures reaching the cecum.

Both preparations were well-tolerated based on patient satisfaction responses, with nearly all patients finding the preparation Tolerable to Very Easy to consume. Most patients (> 70%) who had undergone a prior colonoscopy found FPSS and PEG-SD better than their prior preparation and nearly all would ask for that preparation again for a future colonoscopy. This is an important observation given reports that bowel preparation is a significant deterrent for patient compliance with follow-up colonoscopy.

Walker et al. [4] and Matro et al. [3] observed that PEG-SD was associated with a higher likelihood of electrolyte excursions due to the unbalanced electrolyte composition of the formulation. This observation was not seen here in serum electrolyte changes, perhaps due to the small PEG-SD patient sample and possibly because the PEG-SD patients were much younger than the FPSS patients (by about 10 years).

This study has limitations due to its modest sample size and that the preparation groups were not enrolled in a randomized manner and the local endoscopists were not blinded. These confounders are mitigated somewhat by the addition of blinded central reviewers of the colonoscopy videos, who generally scored FPSS with higher success ratings than PEG-SD. Furthermore, patient lifestyle and water consumption before and during preparation were not recorded. Nevertheless, this study provides a preliminary assessment of the relative efficacy and tolerance of the new sports drink-like flavored FPSS formulation versus PEG-SD and indicates that FPSS may provide better colon cleansing with a similar patient satisfaction profile. This would represent an improvement given that no stimulant laxatives are required with FPSS and the formulation has been demonstrated to be electrolyte balanced [4]. Based on this data, larger safety and efficacy studies of FPSS are warranted.

## Data Availability

The datasets used and/or analyzed during the current study are available from the corresponding author on reasonable request.
